# What motivates Ebola survivors to donate plasma during an emergency clinical trial? The case of Ebola-Tx in Guinea

**DOI:** 10.1371/journal.pntd.0006885

**Published:** 2018-10-17

**Authors:** Maya Ronse, Almudena Marí Sáez, Charlotte Gryseels, Melanie Bannister-Tyrrell, Alexandre Delamou, Alain Guillard, Mustapha Briki, Frédéric Bigey, Nyankoye Haba, Johan van Griensven, Koen Peeters Grietens

**Affiliations:** 1 Department of Public Health, Institute of Tropical Medicine, Antwerp, Belgium; 2 Department of Global Health and Biosecurity, Robert Koch Institut, Berlin, Germany; 3 Centre National de Formation et Recherche de Maferinyah, Forécariah, Guinea; 4 Collecte et Production des PSL, Établissement Français Du Sang Bretagne, Rennes, France; 5 Établissement Français Du Sang Alpes-Méditerranée, Marseille, France; 6 Direction, Établissement Français du Sang Grand-Est, Strasbourg, France; 7 Centre National de Transfusion Sanguine (National Blood Transfusion Centre), Conakry, Guinea; 8 Department of Clinical Sciences, Institute of Tropical Medicine, Antwerp, Belgium; Tulane University School of Public Health and Tropical Medicine, UNITED STATES

## Abstract

**Introduction:**

During the 2014 Ebola Virus Disease (EVD) epidemic, the Ebola-Tx trial evaluated the use of convalescent plasma (CP) in Guinea. The effectiveness of plasmapheresis trials depends on the recruitment of plasma donors. This paper describes what motivated or deterred EVD survivors to donate CP, providing insights for future plasmapheresis trials and epidemic preparedness.

**Methods:**

This qualitative study, part of Ebola-Tx, researched and addressed emergent trial difficulties through interviewing, participant observation and focus group discussions. Sampling was theoretical and retroductive analysis was done in NVivo 10.

**Results:**

Willingness or hesitance to participate in plasma donation depended on factors at the interface of pre-existing social dynamics; the impact of the disease and the consequent emergency response including the trial set-up. For volunteers, motivation to donate was mainly related to the feeling of social responsibility inspired by having survived EVD and to positive perceptions of plasmapheresis technology despite still unknown trial outcomes. Conversely, confidentiality concerns when volunteering due to stigmatization of survivors and perceived decrease in vital strength and in antibodies when donating, leading to fears of loss in protection against EVD, were main deterrents. The dynamic (dis)trust in Ebola Response Actors and in other survivors further determined willingness to participate and lead to the emergence/decline of rumours related to blood stealing and treatment effectiveness. Historic inter-ethnic relations in the health care setting further defined volunteering along socio-economic and ethnic lines. Finally, lack of follow-up and of dedicated care further impacted on motivation to volunteer.

**Conclusions:**

Ebola-Tx was the first trial to solicit and evaluate blood-product donation as an experimental treatment on a large scale in Sub-Saharan Africa. An effective donation system requires directly engaging with emergent social barriers and providing an effective ethical response, including improved and transparent communication, effective follow-up after donation, assuring confidentiality and determining ethical incentives.

## Author summary

During the 2014 West-African Ebola Virus Disease epidemic, the Ebola-Tx clinical trial in Guinea aimed to determine whether the administration of Ebola antibodies from the blood plasma of Ebola survivors could increase Ebola patients’ survival rate. Ebola-Tx was the first trial to solicit and evaluate blood-product donation as an experimental treatment on a large scale in Sub-Saharan Africa. In this qualitative study, part of the Ebola-Tx study, we report on factors of motivation and demotivation influencing Ebola survivors to donate their plasma. Understanding these factors is essential as the successful recruitment of this specific subgroup, providing the therapy itself, directly impacts the effectiveness of such a trial. We show that organizing an effective and ethical donation system requires directly engaging with emerging social barriers at the interface between pre-existing social dynamics, the impact of the disease, and the consequent emergency response including the trial set-up. These results provide insights that can be useful for future plasma trials but also for emergency clinical trials as part of general epidemic preparedness.

## Introduction

The urgency to contain the 2013–2016 West-African Ebola virus disease (EVD) epidemic led to the World Health Organization’s prioritization of the clinical evaluation of convalescent plasma (CP) or whole blood in September 2014 [1–4] as a potential cure, resulting in fast-tracked non-randomized clinical trials in Sierra Leone, Guinea and Liberia [5]. The Ebola-Tx trial in Guinea aimed to determine whether the administration of EVD antibodies from the blood plasma of EVD survivors could increase EVD patients’ survival rate compared to a historical control group receiving only supportive care at the same site. Although the treatment was found to be safe, receiving two consecutive units of 200-250ml CP did not significantly improve the overall survival rate [6].

The effective implementation of such trials depends on the recruitment of EVD survivors as volunteers for plasma donation. Throughout sub-Saharan Africa, even the sampling of small amounts of blood has been associated with considerable fears of ‘blood stealing’ or ‘blood selling’, documented since colonial times [7, 8], leading to reticence for participation in clinical research [8–13]. Reports from Guinea, Sierra Leone and Liberia suggested similar concerns as blood-related rumors were frequently reported during the EVD outbreak [14–17], an example of which being the local suspicion that burial teams of EVD victims “filled their tanks” with the blood of the deceased in exchange for international aid [18]. There were further concerns that the stigmatization of EVD survivors [19, 20] could hinder efforts to recruit survivors to donate plasma [21, 22]. This stigmatization was often related to suspicions of Ebola Treatment Centre workers and survivors having killed patients through mystical means to ensure their own survival in addition to fears of survivors still being infectious. Due to these complexities, scientists were concerned that collection of CP from EVD survivors during the outbreak might not be feasible. Nevertheless, the Ebola-Tx trial recruited 98 survivors, sufficient to provide experimental treatment to all trial patients [23].

This anthropological study was part of the Ebola-Tx trial “*Emergency evaluation of convalescent plasma for Ebola Virus Disease (EVD) in Guinea*” (NCT02342171) and aimed to provide insights into what motivated or deterred EVD survivors to donate convalescent plasma, in order to contribute to the effectiveness of future plasmapheresis trials and outbreak preparedness.

## Methods

### Study site and population

The qualitative research was carried out in both Ebola-Tx trial sites in Guinea–(i) the National Blood Transfusion Centre (NBTC), and (ii) the Médecins Sans Frontières (MSF) run Donka Ebola Treatment Centre–as well as in surrounding areas in Conakry and the three peripheral prefectures of Coyah, Kindia and Dubréka.

#### Ebola-Tx trial

**Fig 1:**
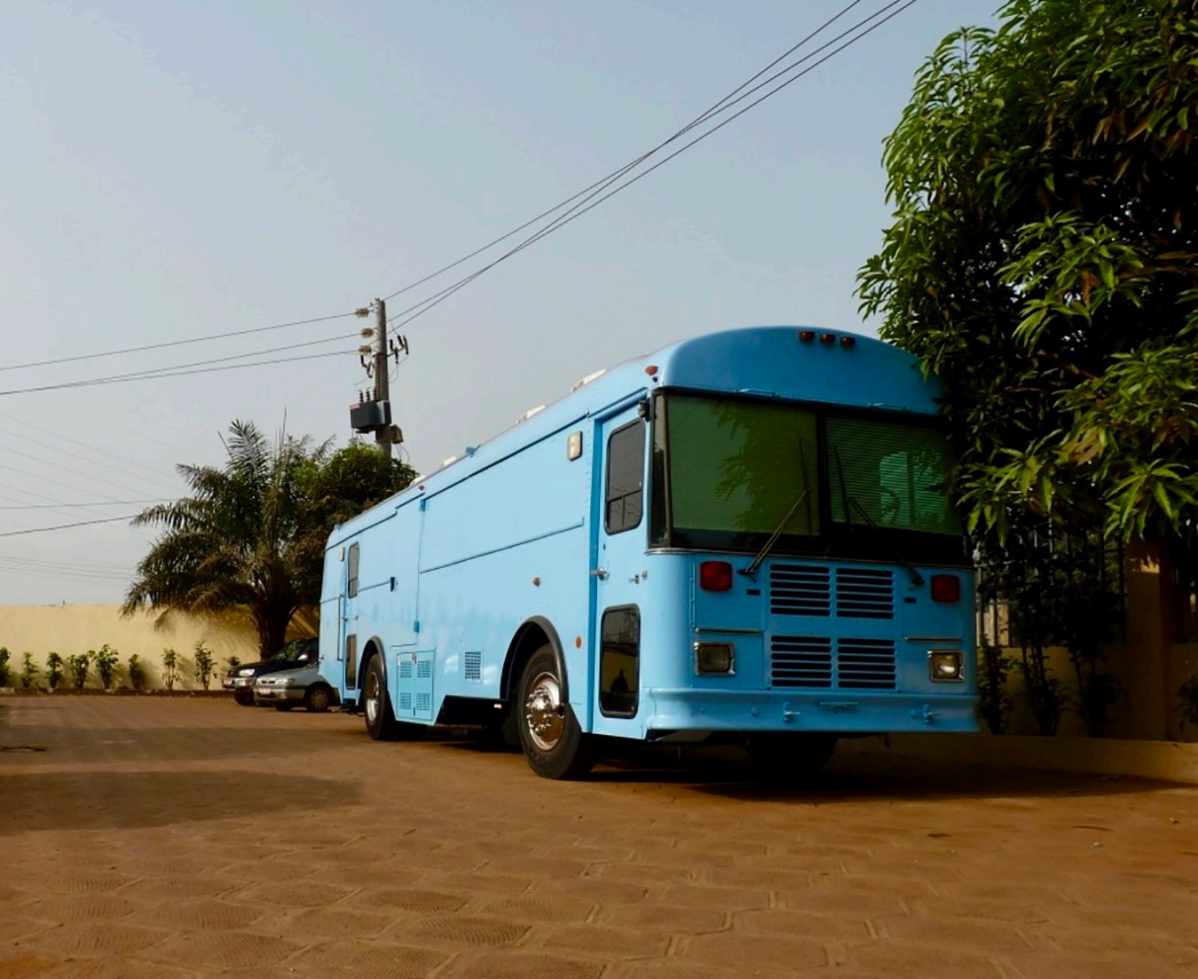
The Plasma mobile.

**Fig 2:**
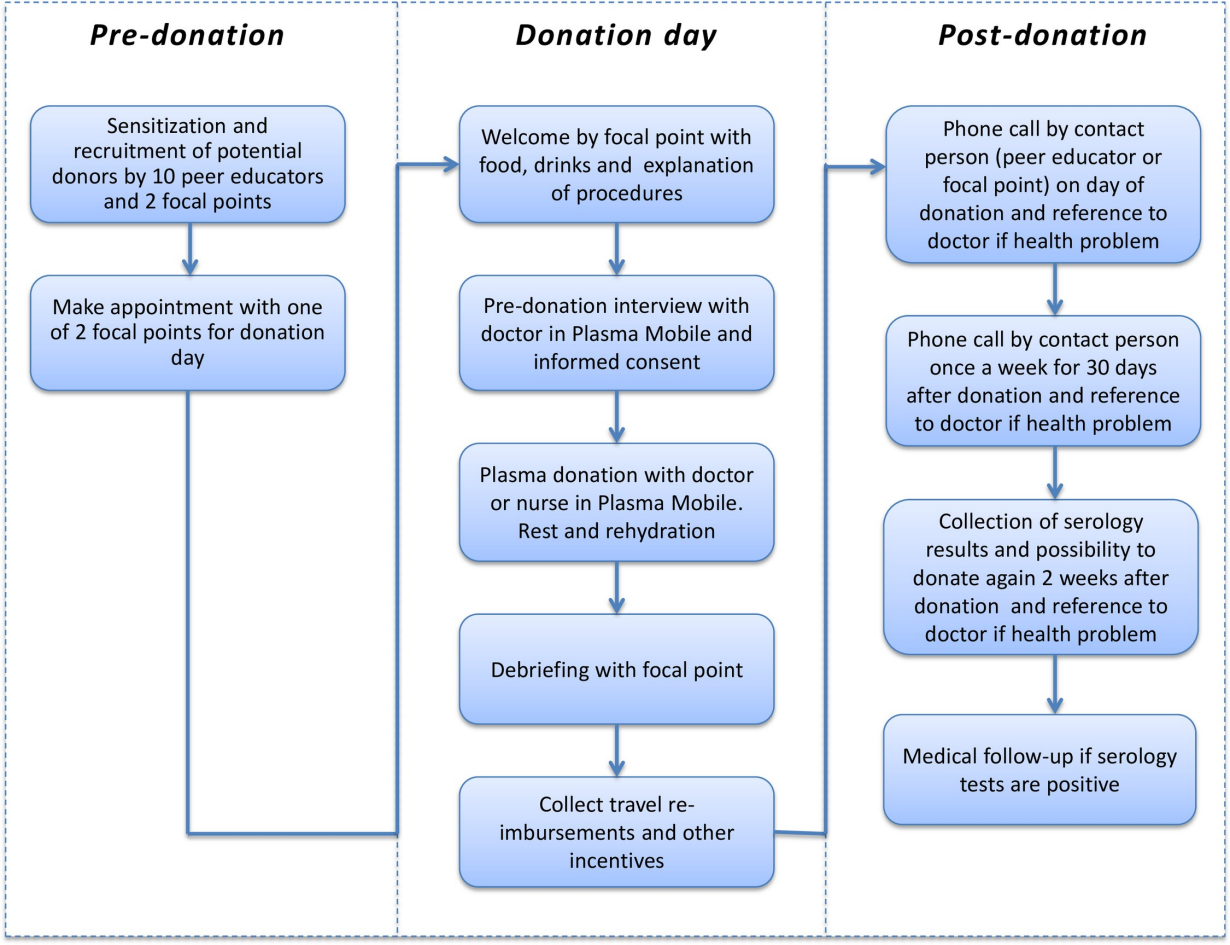
Established donation procedures.

The open-label phase 2/3, non-randomized comparative trial was designed to evaluate the feasibility, safety and efficacy of convalescent plasma (CP) against EVD [5, 6, 23, 24, 25, 26]. During the trial (2015), CP was collected through voluntary donations from EVD survivors and subsequently transfused to confirmed EVD patients at the Ebola Treatment Centre led by MSF. EVD survivors who had recovered from the disease at least 60 days prior to donation were requested to donate plasma. Plasmapheresis was performed in a “Plasma Mobile” at the NBTC (Fig 1) [23], which entailed the removal of whole blood, separation of plasma from red blood cells, and return of red blood cells to the donor’s blood circulation. Donated plasma was then prepared for transfusion, including pathogen reduction, and stored. For donation procedures, see Fig 2.

The Institute of Tropical Medicine (ITM) was the general trial sponsor. Patient care and plasma transfusion was monitored by MSF and plasma donations were managed by the NBTC in collaboration with the “*Association des Personnes Guéries et Affectées d’Ebola en Guinée*” Survivors Association (SA). *Etablissement Français du Sang* (EFS) provided technical capacity building to the NBTC [23]. The Survivors Association was closely involved in the donation system: two focal points, both medical doctors and EVD survivors, mediated between EVD survivors and the trial, led the recruitment of donors, the support to donors on-site and their follow-up post-donation. Ten peer educators were appointed within the SA, under supervision of the focal points, to sensitize potential donors. Including survivors as peer educators was in line with WHO recommendations [27] and the request from the Survivors Association Board during trial preparations. Reasons behind these recommendations included the acknowledgement of the stigma survivors faced and the experience of the HIV epidemic.

### Sampling

Sampling for the study was theoretical (i.e. based on emerging results) [28] (Table 1). Participants were selected based on characteristics such as involvement in the trial, social ties with survivors, professional background, socio-economic status, gender, age, and urban or rural residence.

**Table 1:** Distribution of study participants *.

Categories of participants	Detailed characteristics of informants
**EVD survivors and EVD-affected people**	- Survivors who donated CP - Survivors who did not donate CP - CP recipients who recovered - Relatives from survivors and deceased CP recipients - Members from the Survivors Association in Conakry - Focal points and peer educators working on the trial
**Local staff**	- Guinean NBTC trial staff (doctors, nurses, lab technicians) - Guinean MSF staff (medical staff, health promotion and psychosocial workers, laboratory technicians, WatSan and outreach teams) - Research assistants on the project - Field coordinator - Principal Investigators
**Expatriate staff**	- ITM (the trial coordinator, medical workers, lab experts, trial monitor) - EFS (transfusion specialists)

* This distribution is more complex in reality as people could belong to several categories, e.g. being a survivor, member of the Survivors Association, a CP donor and local MSF staff

### Data collection

Data collection was intermittently carried out between January and September 2015 by two European anthropologists from ITM, in some cases accompanied by an EVD survivor/gatekeeper. This period coincided with phases E and F of the Guinean epidemic [29, 30], right after the highest peak of October-December 2014, characterized by a decrease in cases and a movement of the epidemic to the Lower Guinean region (including the capital).

The position of the researchers and gatekeepers, especially within the context of this epidemic, initially proved challenging in gaining trust of different types of informants. This was gradually overcome through regular visits and constant rapport building. The anthropological team often reflected on and discussed their role in the trial and thus navigated the tension between their critical independence with regards to the trial on the one hand, and, on the other hand, their direct involvement in strategies and activities to improve the effectiveness of the trial.

*Semi-structured in-depth interviews* were carried out using continuously evolving question guides addressing personal experiences with EVD, power relations and social dynamics within the SA, perception and experience of the trial, recommendations for improvement, plasma versus blood perceptions, internal work dynamics, recommendations for further steps of the trial, suggestions for improvement, and rumors pertaining to the trial itself as well as to the Ebola outbreak more generally. *Participant observation* was used at different stages and places linked to trial preparation and implementation, allowing insights into the trial dynamics. *Focus Group Discussions* aimed at eliciting contrasting opinions. Separate group discussions were organized for survivors, donors, trial staff (including doctors) and patients.

### Analysis

Analysis was retroductive and concurrent to data collection. Preliminary data were intermittently analyzed in the field. Temporary results were further confirmed or refuted through constant validity checks until saturation was reached and the data was theoretically supported. Thematic analysis was carried out with NVivo Qualitative Data Analysis software (QSR International Pty Ltd. Cardigan UK).

### Ethical considerations

Oral informed consent was obtained from all adult participants. Considering the sensitive nature of this qualitative research, oral rather than written consent was preferred for a number of reasons: (i) to avoid the high illiteracy in the study population challenging the practical obtaining of written consent; (ii) written consent might seem a breach of confidentiality by the participants; (iii) written consent, requiring a signature and therefore introducing formality in the procedure, affects trust between respondents and researchers and consequently willingness to participate in the study, as conversations usually do not require formal procedures. In addition, this formality introduces bias and hence reduces data quality.

Informed consent was documented in written by the person who obtained the consent and, if present, by a witness. The study, including the use of oral consent, was reviewed and approved by the Institutional Review Board of the ITM (IRB/AB/ac/092 Ref:1098/16) and the Guinean National Ethics Committee for Research in Health–CNERS (131/CNERS/16). Ethical reviews and approval for the Ebola-Tx trial are described in [6].

## Results

23 in-depth interviews and 21 focus group discussions were conducted. Participants in the study included EVD survivors (including CP donors, survivors who did not donate CP, CP recipients, members of the Conakry SA, trial focal points, trial peer educators), relatives of EVD survivors and of deceased patients, and trial staff from the different organizations involved in the trial (see Table 1).

### The meaning of blood, plasma and plasmapheresis technology

Blood was considered sacred and a vital element associated with health. A decrease in volume of blood was perceived to weaken the body and cause (potentially irreversible) sickness, limiting people’s willingness to participate in the trial. Despite the use of plasma instead of blood, and the trial team’s explicit explanation of the difference (e.g. in color, time for recovery, quantities required), participants still perceived the procedure to be “blood” donation. The absence of a word for plasma in the *Susu* language, leading to the use of the word ‘blood’ (‘*wouly*’), contributed to this ambiguity. Donating plasma was thus associated with blood donation, leading to common fears such as the fear of the “unknown” for first-time donors, fear of knowing one’s serological status, fear of the pain, needles, and physical weakening, especially when survivors felt they had not completely recovered from EVD or when they felt they were affected by poor nutrition. Some donors reported needing considerable time to recover. For donors who, conversely, were positively surprised by their smooth recovery, this was mostly linked to their perceptions of the technology being cutting-edge. In addition, the Plasma Mobile and its high-quality equipment was welcomed by survivors as an acknowledgement of previous hardship.

Survivors, especially those with a medical background, perceived the loss of immunity and the increased risk of reinfection and relapse due to the transference of antibodies when donating to be a health risk, as illustrated by the following quote: “*You know it’s the antibodies that helped us recover*. *If you want to weaken our antibodies even more*, *what am I going to do*? *Won’t I become sick again*?*” (EVD survivor*, *IDI)*

### Trust and rumors

One of the main factors influencing decision-making was trust, or the absence of it, in the organizations and people involved.

#### Survivors

The trust in other survivors was a key element for participation. Through their similar experiences, members of the SA shared a common 'survivor’ identity that instigated an implicit feeling of mutual trust and solidarity. This trust and their personal relationships with other survivors were key for potential donors to be convinced by peer educators to participate. Aside from being survivors, most peer educators were health students or professionals, or were respected community members, characteristics that generated trust. However, a lack of transparency within the SA board concerning financial agreements with other organizations, including the Ebola-Tx trial and an ITM-financed sensitization project, led to mistrust, demotivating some survivors to participate.

#### (Inter)national trial staff

The partnership with international organizations, employing both local and international experts, inspired confidence and reassurance. Having international teams onsite (from ITM, MSF, EFS) providing capacity-building and setting a standard of care was considered a sign of quality, which was reported to be an important motivation to donate. Donor interviews indicated a particular appreciation towards Western staff, i.e. as being welcoming, reassuring, and showing personal interest in the donor’s feelings and background. Some survivors built longstanding relationships beyond the scope of the trial with project staff (locals and expatriates), which directly influenced their decision of becoming a (repeat) donor. Despite also being seen as a sign of quality, the presence of international organizations simultaneously raised suspicions and rumors about blood being sold and sent to Western countries. The shipment of plasma samples to France, the difficulty of conveying this information in the informed consent procedure and the consequent ambiguity when procedures were put in practice, was interpreted as confirming on-going rumors.

Trust in the efficacy of the treatment itself was an additional consideration that influenced motivation. Trust plummeted after the temporary suspension of CP donation activities due to logistical difficulties and rumors that some of the first patients had died, yet was restored as soon as a few participating infants and pregnant women–who were known to be less likely to recover from Ebola–did survive.

Historically embedded interethnic relations between the majority Muslim population and the Christian minority (often referred to as “*Forestiers*”) impacted on certain survivors’ willingness to donate, particularly as trial staff mostly in charge of plasma donation identified as Christian. The strong bond between survivors, however, created by the common traumatizing experience of having gone through EVD, usually mitigated the perceived differences in ethnic and religious identities.

### Public acknowledgment and social responsibility in the response

Due to the stigmatization of EVD survivors by neighbors, relatives and more broadly in social and professional networks, the difficulty of dealing with anonymity was decisive for non-participation as it could expose donors as survivors. While some survivors required to be contacted by peers in a confidential way, others demanded openness from peer educators and requested them to sensitize their families, prior to taking the decision to participate. The perception of contributing to patients’ survival and indirect motives such as a sense of patriotism, the feeling of being part of a historic fight against a lethal disease and the feeling of being ‘in debt’ because they survived while others did not, leading to a moral obligation to do something in return, motivated potential donors to participate in the trial.

### Trial set-up

#### Trial procedures

The continuous improvement of trial procedures based on qualitative research contributed to increasing acceptability of the trial. Examples of this flexible approach were the adoption of more effective and accessible travel reimbursements to allow very poor survivors, unable to advance transportation fees, to attend; and the joint development of information tools provided to survivors on the relevance of plasma donations, including guidelines for peer educators and staff containing information about the trial, its donation procedures and FAQ.

#### Post-donation follow-up

The emergency context and aim for a cure distracted the trial team from the need for follow-up procedures. While offered care during follow-up was a motivating factor among networks of trial participants with a positive experience in this respect, some donors experienced long delays in the delivery of blood test results and care for additional health problems, which acted as a demotivating factor for repeat donors and other survivors.

#### Location of the trial sites

The NBTC was part of the Donka Hospital complex where the Ebola Treatment Centre was also situated, but isolated from the main hospital buildings. Many survivors perceived this separate location as suspicious as it was initially the link with the Ebola Treatment Centre where they were once successfully treated for EVD that legitimized the CP donation. For some survivors and/or their relatives, however, returning to “Donka” was “tempting faith” after the “miracle” of survival.

#### Incentives

Specific trial benefits also drove participation, such as the provision of phone credit, reimbursement for transport costs and free testing for infections like HIV, hepatitis (B and C) and syphilis. Access to the trial site, however, was difficult for people living in remote areas given the extreme poverty in this region. Additional assistance from the trial could have mitigated this according to some informants, for example by providing nutrition, especially as the donation of blood products was perceived to weaken the body.

## Discussion

The Ebola-Tx trial was remarkable for being the first to evaluate convalescent plasma on a large scale in Sub-Saharan Africa and for being amongst the first to solicit blood-product donation on a large scale as an experimental treatment in this region. [31, 32] The estimated proportion of donors contributing to the study represented 18% (98/547) of the EVD survivors residing in the Conakry area at the time of the study. [23] Voluntary blood-product donation in Guinea Conakry constitutes an uncommon yet problematic and contested issue, which needs to be understood in relation to the historical, sociocultural and political context of Guinea. French colonization, subsequent dictatorships and associated repression of and violence towards certain ethnic groups, corruption, distrust of the political establishment, wariness towards foreign help, and country-wide interethnic conflicts, exacerbated by the EVD outbreak, profoundly shaped the Guinean population’s perceptions of the response. [33–40] It is in this context that EVD survivors were required to come forth for plasma donation.

**Fig 3:**
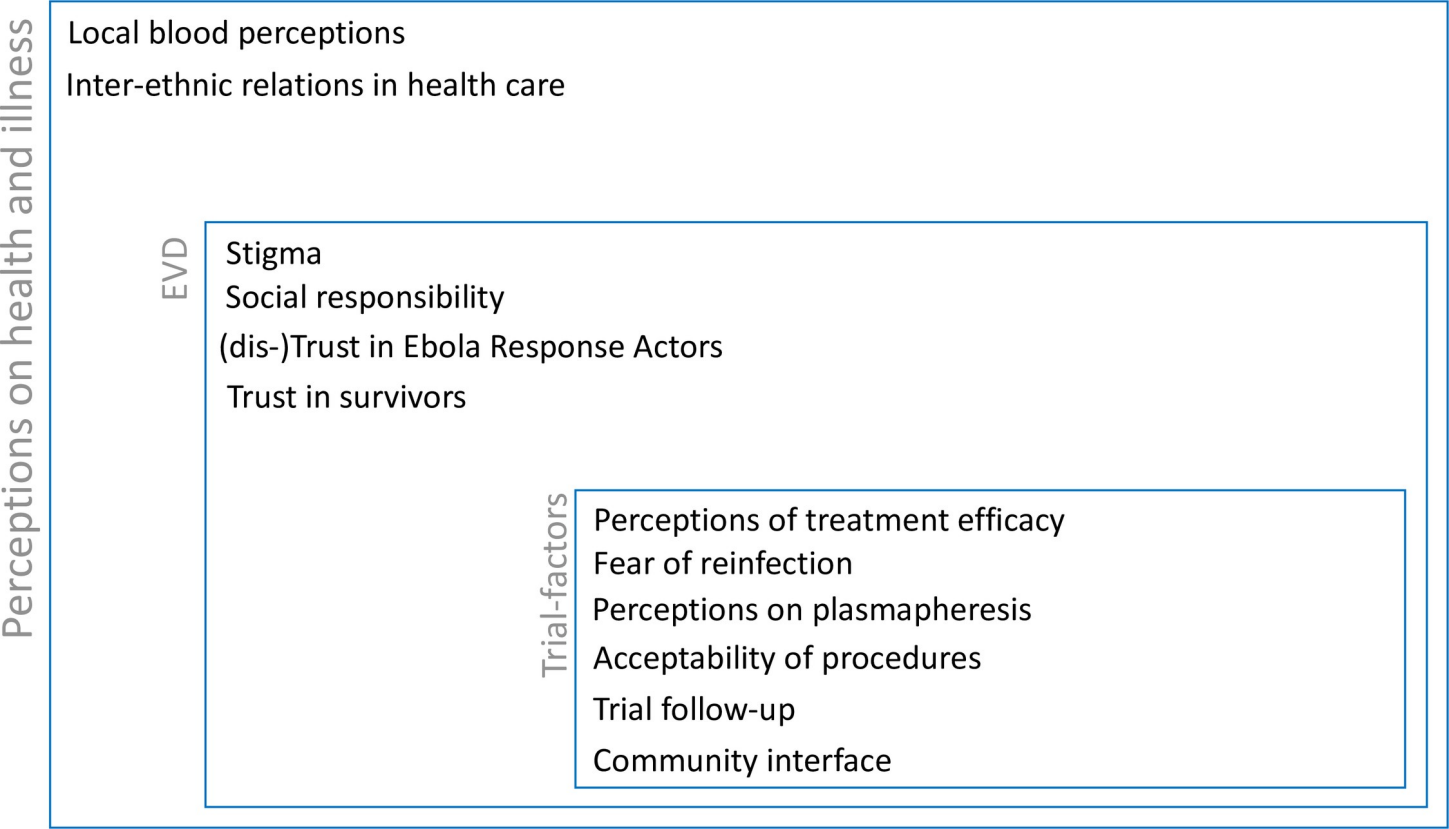
Summary of factors influencing the decision-making of participation in CP donation.

As such, the willingness or hesitance of survivors to become donors depended on a combination of factors linked to health and illness perceptions occurring at the interface of the characteristics of the EVD epidemic and of the consequent response, including the trial, itself (Fig 3).

Constituting a blood-related product, plasma was still perceived as *blood* and did not avoid pre-existing sensitivities related to the symbolic value of (whole) blood such as difficulties related to blood sampling or blood-related rumors. Additionally, the fear of EVD reinfection and relapse after a decrease in antibodies level, as well as the perceived consequences for their general health, indicate participants’ perceptions of both the protection and the strength their blood signifies. EVD outbreak-specific factors included the interplay and contrasts between survivors’ experiences of stigmatization in the broader community on the one hand and the trust and solidarity among survivors on the other hand. Emerging rumors and trust in the organizations and staff involved in the trial further affected motivation to participate. The ambiguity between both historically rooted and epidemic-related mistrust, and participants’ feelings of social responsibility and trust in foreign interventions, was also described in other EVD trial settings. [17]

Although the context of Guinea and EVD is highly specific, literature on motivating factors for donation of whole blood in low and middle income countries and of plasma in Western countries [12, 21, 22, 41–44] reports strong similarities with the Ebola-Tx trial, including the relevance of comprehensive information on trial procedures (e.g. during sensitization) as well as on the destination and use of blood samples, perceived benefits of participation in the form of incentives, and trial staff’s attention to psychological well-being.

Reasons for participation in medical research in general have been widely documented. [8, 45–59] A specificity of this intervention, however, lies in the recruitment of survivors as participants for plasma donation and not as trial patients, leading to new ethical questions surrounding the use of CP before trial implementation [3, 60]. To the best of our knowledge, no ethical guidelines existed for survivors providing an experimental product before the start of a trial. This gap was partially filled by the conception of WHO guidelines on the use of Convalescent Blood and Plasma [3, 27, 61, 62] that advised considering the CP donors as research participants, similar to the CP recipients. This logic was followed in Ebola-Tx, hereby extending the risks and benefits from EVD patients to the CP donors [60].

### Study limitations

Time limitations, security measures, initial people’s distrust and financial and political incentives for respondents as part of the response to the epidemic did not always allow for the researchers’ immersion in the study setting as is usual with ethnographic research methods. This limited access to sensitive information and informants, which limited the scope and depth of the collected data.

## Conclusion

Actively engaging with, responding to and following up participants, especially when concerning vulnerable populations such as EVD survivors enrolled as plasma donors, should be integral to the trial design. Effective and ethical donation systems, emergency trials, and even trials in low-resource settings more broadly, require directly engaging with emergent factors that occur at the interface between pre-existing social dynamics, the impact of the disease, and the consequent (emergency) response including the trial set-up. We advocate for the integration of methodologies to face emergent factors into trial designs from the start.
